# Alleviation of Acute Heat Stress in Broiler Chickens by Dietary Supplementation of Polyphenols from Shredded, Steam-Exploded Pine Particles

**DOI:** 10.3390/microorganisms13020235

**Published:** 2025-01-22

**Authors:** Akshat Goel, Chris-Major Ncho, Chae-Mi Jeong, Vaishali Gupta, Ji-Young Jung, Si-Young Ha, Jae-Kyung Yang, Yang-Ho Choi

**Affiliations:** 1 Division of Animal Science, Gyeongsang National University, Jinju 52828, Republic of Korea; genesakshat@gnu.ac.kr (A.G.); chrismajor.ncho@usys.ethz.ch (C.-M.N.); wjdcoa@gnu.ac.kr (C.-M.J.); vaishali2020@gnu.ac.kr (V.G.); 2 Institute of Agriculture and Life Sciences, Gyeongsang National University, Jinju 52828, Republic of Korea; charmhanjjy@nate.com (J.-Y.J.); jkyang@gnu.ac.kr (J.-K.Y.); 3 Division of Applied Life Sciences (BK21 Four Program), Gyeongsang National University, Jinju 52828, Republic of Korea; 4 Department of Environmental Materials Science, Gyeongsang National University, Jinju 52828, Republic of Korea; hellohsy2@gmail.com

**Keywords:** acute heat stress, broilers, cecum metagenome, gene expression, polyphenols from steam-exploded pine particles

## Abstract

Reducing the detrimental effects of heat stress (HS) in poultry is essential to minimize production losses. The present study evaluates the effects of dietary polyphenols prepared from underutilized wood byproducts on the growth, gut health, and cecal microbiota in broilers subjected to acute heat stress (AHS). One hundred eight one-day-old Indian River broilers were fed with 0%, 0.5%, or 1% polyphenols from shredded, steam-exploded pine particles (PSPP) in their diet. On the 37th day, forty birds were equally distributed among four groups containing either a control diet at thermoneutral temperatures (NT0%) or AHS temperatures with 0% (AHS0%), 0.5% (AHS0.5%), and 1% (AHS1%) PSPP-supplemented diets. The temperature in the NT room was maintained at 21.0 °C, while, in the AHS room, it was increased to 31 °C. AHS negatively influenced performance parameters and increased rectal temperature (RT) in broilers. The AHS0% group showed a higher expression of NOX4, HSP-70, and HSP-90 genes, while the expression was lower in PSPP-supplemented birds. In the jejunum, mRNA expression of *SOD* was increased in all the birds under AHS compared to NT. The expression of the *CLDN1* and *ZO2* genes was increased in AHS0%, while that of the *ZO1* and *MUC2* genes was increased in PSPP-supplemented birds. HS tends to increase *TLR2* and *TLR4* gene expression in chickens. The significantly modified genera were *Bariatricus*, *Sporobacter*, *Sporanaerobacter*, and *Natranaerovirga*. Concludingly, AHS negatively influences the performance parameters, RT, stress, gut-health-related genes, and pathogenic penetration, but PSPP supplementation reduces its bad impact by overcoming the stress and gut-health-related genes, increasing favorable bacterial abundance and reducing pathogenic penetration in chickens.

## 1. Introduction

Global warming is becoming a severe threat around the world and its obvious effects in the form of heat stress (HS) have been observed on livestock production. Poultry is among those industries that are facing detrimental effects of HS, leading to antioxidant modulation, panting behavior, challenged immunity, enhanced rectal temperature (RT), retarded growth performance, and enhanced mortality rates in chickens [1,2,3,4]. Acute heat stress (AHS) in broilers has been known to induce oxidative stress [5]. Further, plasma metabolites such as cholesterol, glucose, and thyroid hormones were disturbed under AHS [6]. The growth of the broilers is hindered, along with inflammation developing in the intestine [7]. In general, gut health plays a crucial role due to its active role in nutrient absorption and protection against pathogen invasion. Epithelial cells and tight junctions maintain the intestinal barrier’s integrity. Through the formation of a mucus layer, mucin inhibits the pathogen penetration [8]. However, by connecting tight junctions and closing the extracellular gaps, zona occludens, claudin, and occludin function as a shield [9]. HS negatively influences the intestinal integrity causing leaky gut in broilers [10]. Antioxidants and stress-related genes are among those that are modified under HS conditions [11] and can be useful to assess the impact of HS in chickens.

Dietary feed additives rich in natural compounds and substances have the capability to mitigate HS effects via antioxidant defense mechanism [12,13]. Nonetheless, the highly competitive nature of the poultry industry necessitates the use of economical stuff as feed additives. One potential solution could be the extraction of valuables from the inexpensive byproducts of the wood industry and using them in broiler ration. In light of the above information, steam-exploded pine particles (SPP) have been previously explored to modulate performance and gut microbiota in chickens [14]. Recent advances in technology allow value addition by separating solubles from SPP (SSPP) to mitigate heat stress [15]. Further, for the present study, polyphenols extracted from shredded, steam-exploded pine particles were used. Polyphenols are the fourth most abundant components in plant tissue after celluloses, hemicelluloses, and lignin. Owing to their antimicrobial and antioxidative properties, their supplementation improves performance parameters in chickens [12]. Previous studies have demonstrated the beneficial effects of phenolic compounds from various plant sources on the growth performance, microbiota, and antioxidant status in microbial or heat challenged monogastric animals [16,17].

The usage of wood powder as shredded, steam-exploded pine particles (SPP) and solubles from SPP (SSPP) has already been investigated to alleviate the harmful effects of acute heat stress (AHS) in chickens [15,18]. However, the role of polyphenols from pine wood sources has not yet been explored. Hence, the purpose of this study is to evaluate the effect of dietary supplementation with polyphenols from shredded, steam-exploded pine particles (PSPP) extracted from SPP on the antioxidant capacity, gut health, and cecal microbiota in acute-heat-stressed broiler chickens. We hypothesize that dietary PSPP supplementation may mitigate the AHS effects by modifying the cecal microbiota, antioxidant, and gut-health-related genes in chickens.

## 2. Materials and Methods

The birds used in this study were raised in the animal research facility of Gyeongsang National University, Republic of Korea.

### 2.1. Preparation of the Polyphenolic Extract

The preparation of PSPP was achieved through a series of steps. Initially, pinewood chips approximately 2 × 2 × 0.5 cm^3^ in dimensions were exploded with steam at 200 °C for 11.5 min, as explained previously in our previous studies [13,14,18]. PSPP was then further extracted using ethanol.

### 2.2. Experimental Birds and Housing

**Figure 1:**
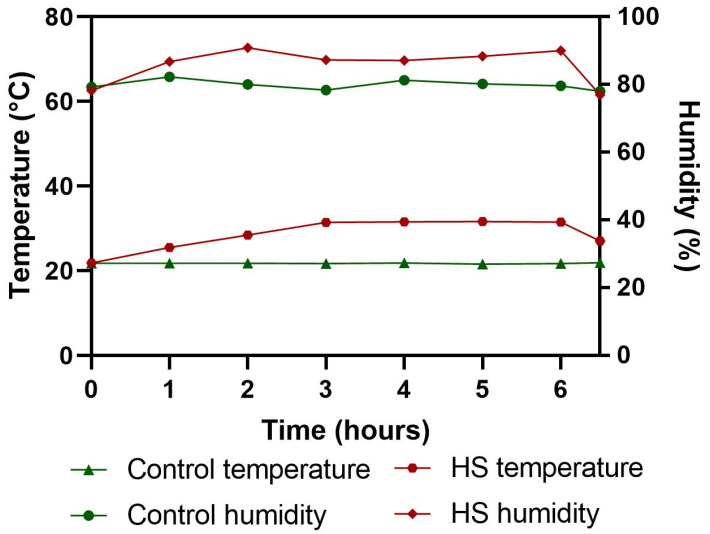
The temperatures of the thermoneutral and heat stress room during the acute heat stress study.

In total, one hundred eight 1-day-old Indian River broiler chicks were obtained from a commercial chick producer. The chicks were obtained and distributed as hatched without sexing. The poultry house was disinfected using orthophenylphenol (#fumagri OPP, Kersia, Dinard Cedex, France) 36 h before the arrival of birds. After arrival, the chicks were individually weighed, tagged, and assigned to one of three dietary treatment groups and supplemented with three levels of PSPP (0, 0.5, and 1%) in addition to the basal commercial feed (Supplementary Table S1). The birds were raised in a closed, thermally controlled environment. The rooms were equipped with exhaust fans to maintain air quality. White LED ceiling lights were used and a lightening regime of 23 h light and 1 h darkness was followed as stated in our previous study [19]. The brooding temperature was maintained at 34 °C during the first three days and was gradually decreased to reach 21 °C on day 27 of age. This temperature was maintained until day 36 of age. Feed was provided ad libitum but measured before offering. Nipple-style drinkers were used to supply water, but water intake was not measured. A total of 40 birds that were close to the average body weight (BW) on the 37th day of age were chosen and equally divided into four groups of five duplicate cages, each with two birds. The cage dimensions were: 90 cm (length) × 70 cm (width) × 45 cm (height). The group description is as follows: NT0%, control diet without any PSPP supplementation kept at a thermoneutral temperature (21.0 °C); AHS0%, control diet (Supplementary Table S1) without PSPP supplementation kept at an AHS temperature; AHS0.5%, supplemented with 0.5% PSPP kept at an AHS temperature; and AHS1%, supplemented with 1% PSPP kept at an AHS temperature. In the AHS room, the temperature was gradually increased hourly to achieve 31 °C in three hours. The peak temperature (31 °C) was maintained for the next three hours. The temperature variations and AHS protocol for the rooms are displayed in Figure 1.

### 2.3. Sampling and Data Collection

At the beginning (before HS) and end (after AHS) of the heat exposure, the individual body weight (BW) of the birds was recorded. The difference in BW was calculated using the below mathematical equation:

PDBW (%) = [(final body weight − initial body weight)/initial body weight] × 100

Further, the residual feed in the feeders was measured to calculate the feed intake (FI) of the birds, as shown below:

FI (g) = feed offered − feed remaining in the feeders

Similarly, rectal temperature (RT) was recorded before and after heat exposure using a digital thermometer (HI 91610, Hanna instruments Inc., Padova, Italy). The probe was inserted approximately 3 cm inside the cloaca and the temperature was recorded when the reading was stabilized. The difference in RT (ΔT) was calculated based on temperatures recorded before and after heat exposure.

After the completion of the AHS protocol, sample collection was performed on six birds per treatment. The birds were randomly chosen and slaughtered using a carbon dioxide chamber. Blood samples (5 mL) from each bird were collected in heparinized vacutainers via a cardiac puncture. Thereafter, plasma was collected via centrifugation at 2000× *g* for 10 min at 4 °C. The collected plasma samples were then stored at −20 °C until use.

Jejunum and cecal samples were collected and snap frozen in liquid nitrogen, while immune organs (liver without gallbladder, spleen, and bursa of Fabricius) were isolated and the absolute organ weight was recorded. The relative organ weight was calculated as:

Relative organ weight (%) = (absolute organ weight/body weight of the bird) × 100

### 2.4. Plasma Biochemical Analysis

Plasma metabolite concentrations (glucose, total protein, triglycerides, and total cholesterol) were evaluated using dry-slide technology in a VetTest chemistry analyzer (IDEXX Co., Ltd., Westbrook, ME, USA) [4]. The user manual was followed for the stepwise protocol.

### 2.5. Quantitative Real-Time PCR (qPCR) for Jejunal mRNA Expression

The manual method using Trizol as a reagent was used to isolate total RNA from RNA samples. Briefly, approximately 50 mg of jejunum tissue was homogenized with 800 µL of Trizol™ reagent (Thermo Fisher Scientific, Waltham, MA, USA). Further steps were carried out using chloroform and isopropyl alcohol. Finally, chilled ethanol was used for pellet washing and deionized water was used to dilute the RNA. To determine the concentration and purity of RNA samples, a nanodrop (Thermo Scientific, Waltham, MA, USA) was used and absorption was read at 260 and 280 nm wavelengths. The obtained RNA samples were used to further synthesize cDNA using the Verso cDNA synthesis kit (Thermo Fisher Scientific, Waltham, MA, USA). The relative expression of stress markers and gut-health-related genes (Supplementary Table S2) was assessed using StepOnePlus™ real-time PCR systems (Life Technologies, Carlsbad, CA, USA). The reaction consisted of cDNA, Power SYBR™ green PCR master mix (Life Technologies, Carlsbad, CA, USA), and 10 pmol of forward and reverse primers for a specific gene. Two housekeeping genes (*glyceraldehyde-3-phosphate dehydrogenase (GAPDH) and β-actin*) were used for the Ct value normalization. The relative expression of each gene was then determined using the 2^−ΔΔct^ algorithm.

### 2.6. Metagenome Investigation

A DNeasyPowerSoil Kit (Qiagen, Hilden, Germany) was used to extract genomic DNA from the cecum. The DNA quantification was performed using Quant-IT PicoGreen (Invitrogen, Waltham, MA, USA). Finally, a 16S metagenomic sequencing library was constructed using Herculase II Fusion DNA Polymerase Nextera XT Index Kit V2 (Illumina, San Diego, CA, USA). The library was then sequenced on the Illumina platform (Macrogen, Inc., Seoul, Republic of Korea). Quality profiling, adapter trimming, and read filtering was done using the fastp platform [20] and sequences in the range of 400–500 bp were used. Sequence assembly was conducted using the FLASH (v1.2.11) (Johns Hopkins University, Baltimore, MD, USA) software [21]. The number of operational taxonomic units (OTUs) with a 97% sequence identity criterion was calculated using the CD-HIT-EST program [22]. The taxonomic similarity against the reference database (NCBI 16S Microbial) was calculated by using the BLAST+ (v2.9.0) program (National Library of Medicine, Rockville Pike, Bethesda, MD, USA) [23]. In case of identical coverage, < 85% was considered undefined. Using the QIIME (v1.9) (Northern Arizona University, Flagstaff, AZ, USA) software, OTU abundance was assessed and the microorganisms’ taxonomic details were obtained. Alpha diversity was measured in terms of microbiological species diversity and homogeneity using the OTU, Chao1, Shannon, Good’s coverage, and Gini–Simpson indices. The beta diversity was calculated using the unweighted/weighted UniFrac distances.

### 2.7. Statistical Analysis

The growth parameters were analyzed using ANCOVA. Other parameters such as RT, organ weights, plasma biochemicals, and relative mRNA expression were analyzed using a general linear model (GLM) procedure for the one-way ANOVA. Significances among treatments were determined using a Duncan’s multiple range test. Planned contrasts were performed to compare NT0% vs. AHS0%; NT0% vs. AHS0.5%; NT0% vs. AHS1%; AHS0% vs. AHS0.5%; AHS0% vs. AHS1%; AHS0% vs. AHS0.5% and AHS1%; and NT0% vs. AHS0%, AHS0.5%, and AHS1%. The mean ± standard error of the mean (SEM) was used to express the data, and statistical differences were defined as *p* < 0.05, unless otherwise stated. Alpha diversity and taxonomic abundance of the cecal microbiota in terms of phylum and genus were performed using the Kruskal–Wallis test with the Bonferroni correction. A principal coordinate analysis (PCoA) based on unweighted and weighted UniFrac differences was used to display the beta diversity. Statistical significance in terms of beta diversity was evaluated using PERMANOVA. The data obtained in this study were analyzed using IBM SPSS Statistics package 25.0 (IBM software, Chicago, IL, USA) and the SAS software version 9.4 (SAS Institute Inc., Cary, NC, USA). The Prism-GraphPad software version 8 (Graphpad Software, San Diego, CA, USA) was used to construct the graphs.

## 3. Results

The effect of AHS on the growth performance parameters of broilers fed different concentrations of PSPP in their diet is presented in Table 1. The ANCOVA analysis revealed that FBW, PDBW, and FI differed (*p* < 0.05) among treatment groups. The FBW was positively correlated with the IBW (*p* < 0.05). PDBW and FI had no relation with IBW. The contrast analysis revealed that PDBW and FI were negatively influenced under HS without having diet effects.

The RT before starting the AHS experiment was similar (*p* > 0.05) among treatments. However, at the end of AHS, birds under the AHS condition (AHS0%, AHS0.5%, and AHS1%) had a significantly higher RT compared with those at the thermoneutral temperature (NT0%). The difference in the initial and final temperature represented by ΔT revealed that AHS0.5% had the highest while AHS1% had the lowest increment in the RT (ΔT; *p* < 0.001) at the finishing time of the HS experiment (Table 2).

Table 3 shows the absolute and relative organ weights. The absolute and relative weight of the liver, spleen, and bursa of Fabricius showed no significant differences across the treatments (*p* > 0.05).

Various plasma metabolites such as glucose, total protein, triglycerides, and cholesterol were significantly indifferent (*p* > 0.05) among the groups. However, the contrast analysis revealed that HS tended to decrease triglyceride levels in chickens (NT0% vs. AHS0%, AHS0.5%, and AHS1%, *p* = 0.067). Additionally, Table 4 indicates that PSPP-supplemented heat-exposed birds had lower triglyceride levels compared to control birds at the thermoneutral temperature (NT0% vs. AHS0.5%, *p* = 0.037; NT0% vs. AHS1%, *p* = 0.080).

**Figure 2:**
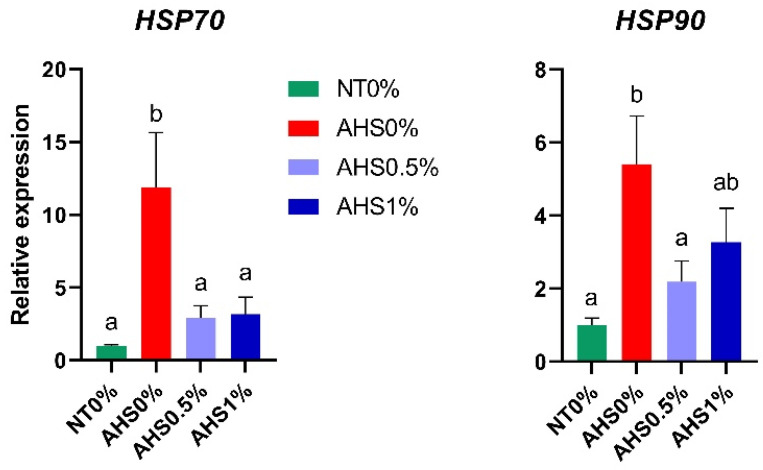
Effect of dietary polyphenol extract supplementation on the expression of heat-shock-protein-related genes in the jejunum of broiler chickens. Chickens were fed diets containing 0% (control), 0.5%, and 1% polyphenolic extract from the 1st day to the 36th day of age. On the 37th day, birds were either kept at a thermoneutral temperature (21.0 °C) and provided with a control diet (NT0%) or heat-stressed at 31.0 °C for six hours and supplemented with 0% (AHS0%), 0.5% (AHS0.5%), and 1% (AHS1%) polyphenolic extract in their diet. The data show the mean ± SEM (*n* = 6). a, b: different letters indicate significant differences (*p* < 0.05). Abbreviations: NT, normal temperature; AHS, acute heat stress.

Figure 2 displays the jejunal mRNA expression of the *heat shock protein (HSP)-70* and *-90*. The expression of both the *HSP* genes (*HSP-70* and *-90*) was increased (*p* < 0.05) in AHS0% compared to NT0%. Additionally, the contrast analysis (Table 5) revealed that heat exposure (NT0% vs. AHS0%, AHS0.5%, and AHS1%) significantly increased the gene expression of *HSP-70* (*p* = 0.024) and *HSP-90* (*p* = 0.011).

**Figure 3:**
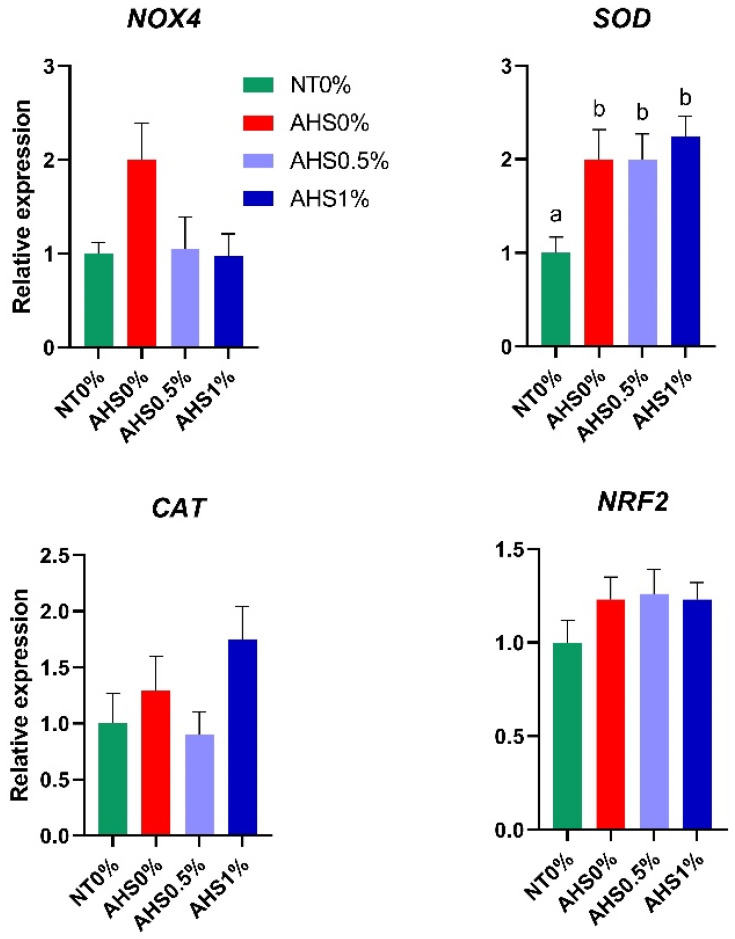
Effect of dietary polyphenol extract supplementation on the expression of antioxidant-related genes in the jejunum of broiler chickens. Chickens were fed diets containing 0% (control), 0.5%, and 1% polyphenolic extract from the 1st day to the 36th day of age. On the 37th day, birds were either kept at a thermoneutral temperature (21.0 °C) and provided with a control diet (NT0%) or heat-stressed at 31.0 °C for six hours and supplemented with 0% (AHS0%), 0.5% (AHS0.5%), and 1% (AHS1%) polyphenolic extract in their diet. The data show the mean ± SEM (*n* = 6). a, b: different letters indicate significant differences (*p* < 0.05). Abbreviations: NT, normal temperature; AHS, acute heat stress.

The expression of antioxidant- and reactive-oxygen-species-related genes such as *CAT*, *SOD*, *NOX4*, and *NRF2* is presented in Figure 3. The expression of *SOD* was increased (*p* < 0.05) in all the HS treatment groups (AHS0%, AHS0.5%, and AHS1%) compared to NT0%. No significant difference (*p* > 0.05) was observed in the expression of *CAT*, *NOX4*, and *NRF2* genes. The contrast analysis revealed that the expression of the *NOX4* gene was increased in AHS0% compared to NT0% (Table 5). However, its expression was reduced (*p* < 0.05) in AHS0.5% and AHS1% compared to AHS0%.

**Figure 4:**
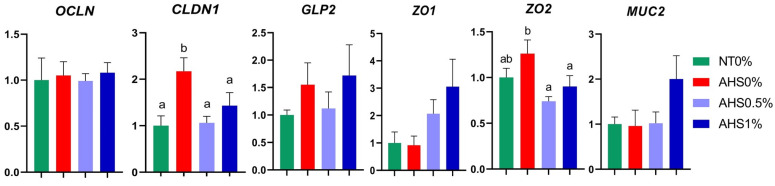
Effect of dietary polyphenol extract supplementation on the expression of gut-health-related genes in the jejunum of broiler chickens. Chickens were fed diets containing 0% (control), 0.5%, and 1% polyphenolic extract from the 1st day to the 36th day of age. On the 37th day, birds were either kept at a thermoneutral temperature (21.0 °C) and provided with a control diet (NT0%) or heat-stressed at 31.0 °C for six hours and supplemented with 0% (AHS0%), 0.5% (AHS0.5%), and 1% (AHS1%) polyphenolic extract in their diet. The data show the mean ± SEM (*n* = 6). a, b: different letters indicate significant differences (*p* < 0.05). Abbreviations: NT, normal temperature; AHS, acute heat stress.

The expression of gut-health-related genes such as *OCLN*, *CLDN1*, *GLP2*, *ZO1*, *ZO2*, and *MUC2* is presented in Figure 4. The expression of *CLDN1* was increased (*p* < 0.05) in AHS0% compared to its counterparts. Similarly, the expression of *ZO2* was also increased (*p* < 0.05) in AHS0% compared to AHS0.5% and AHS1% but was similar in NT0%. No significant difference (*p* > 0.05) was observed in the expression of *OCLN*, *GLP2*, *ZO1*, and *MUC2* genes among treatment groups. The contrast analysis revealed that *ZO1* expression was increased (*p* < 0.05) in AHS1% compared to NT0% and AHS0% (Table 5). Additionally, among heat-exposed birds, its expression showed a significant increase (*p* < 0.05) in the PSPP-supplemented birds (AHS0% vs. AHS0.5% and AHS1%). The expression of *MUC2* was increased (*p* < 0.05) in PSPP-supplemented birds when they were exposed to HS (AHS0% vs. AHS1%; Table 5).

**Figure 5:**
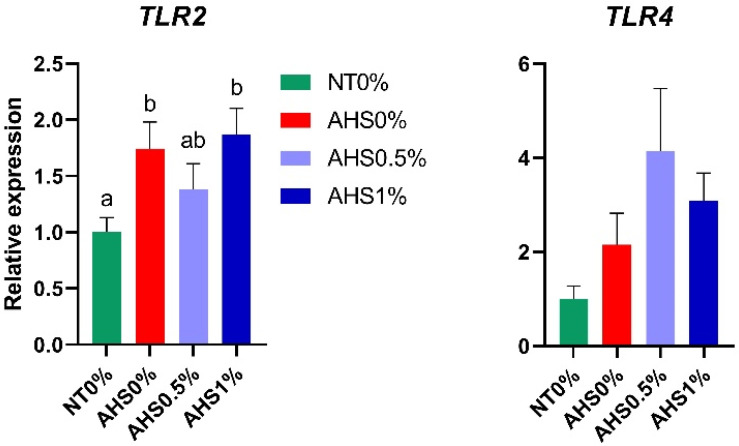
Effect of dietary polyphenol extract supplementation on the expression of immunity-related genes in the jejunum of broiler chickens. Chickens were fed diets containing 0% (control), 0.5%, and 1% polyphenolic extract from the 1st day to the 36th day of age. On the 37th day, birds were either kept at a thermoneutral temperature (21.0 °C) and provided with a control diet (NT0%) or heat-stressed at 31.0 °C for six hours and supplemented with 0% (AHS0%), 0.5% (AHS0.5%), and 1% (AHS1%) polyphenolic extract in their diet. The data show the mean ± SEM (*n* = 6). a, b: different letters indicate significant differences (*p* < 0.05). Abbreviations: NT, normal temperature; AHS, acute heat stress.

Figure 5 shows the mRNA expression of genes such as *TLR2* and *TLR4*. The expression of *TLR2* was increased (*p* < 0.05) in AHS0% and AHS1% compared to NT0%. The expression of *TLR4* was significantly indifferent among treatments. The results obtained from the contrast analysis showed that the expression of *TLR4* was increased (*p* < 0.05) in HS0.5% compared to NT0% (Table 5). Furthermore, HS tended to increase (*p* < 0.05) the expression of the *TLR2* and *TLR4* genes compared to birds kept at the thermoneutral temperature (NT0% vs. AHS0%, AHS0.5%, and AHS1%; Table 5).

**Figure 6:**
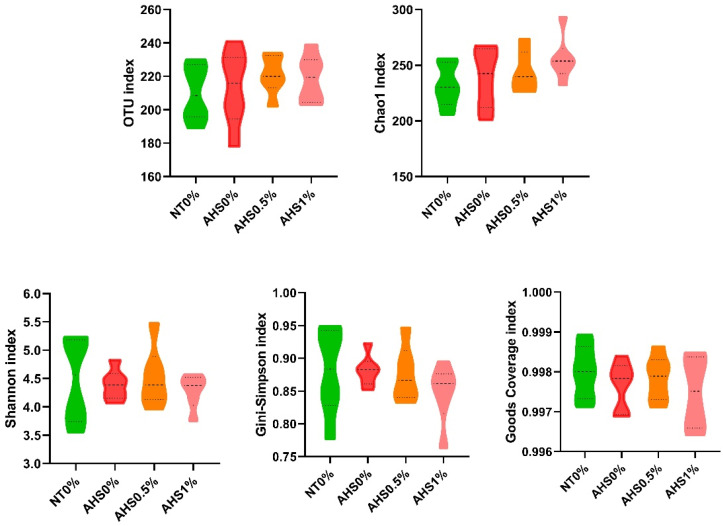
Effects of dietary polyphenol extract supplementation on the alpha diversity indices in the chicken cecum. Chickens were fed diets containing 0% (control), 0.5%, and 1% polyphenolic extract from the 1st day to the 36th day of age. On the 37th day, birds were either kept at a thermoneutral temperature (21.0 °C) and provided with a control diet (NT0%) or heat-stressed at 31.0 °C for six hours and supplemented with 0% (AHS0%), 0.5% (AHS0.5%), and 1% (AHS1%) polyphenolic extract in their diet. The data show the mean ± SEM (*n* = 6). Abbreviations: NT, normal temperature; AHS, acute heat stress.

To access the alpha diversity in the chicken cecum, community richness (OTUs and Chao1) and diversity (Shannon, Gini-Simpson, and Good’s coverage indices) were analyzed (Figure 6). The alpha diversity indices in the cecum of the PSPP-supplemented and heat-exposed broilers did not differ significantly.

**Figure 7:**
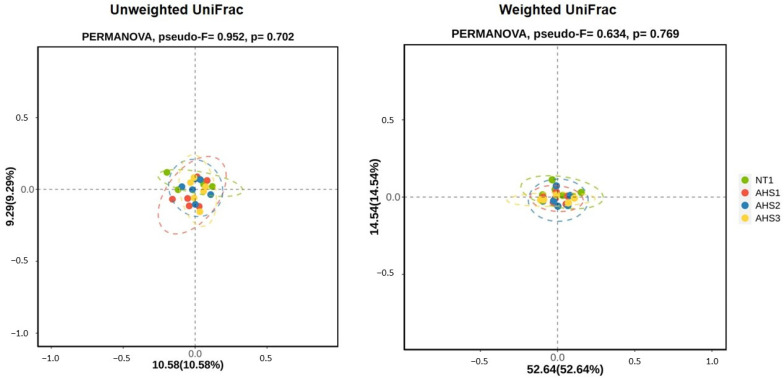
Effects of dietary polyphenol extract supplementation on the beta diversity indices analyzed through unweighted and weighted UniFrac distances based on principal coordinate analysis (PCoA) in the chicken cecum. Chickens were fed diets containing 0% (control), 0.5%, and 1% polyphenolic extract from the 1st day to the 36th day of age. On the 37th day, birds were either kept at a thermoneutral temperature (21.0 °C) and provided with a control diet (NT0%) or heat-stressed at 31.0 °C for six hours and supplemented with 0% (AHS0%), 0.5% (AHS0.5%), and 1% (AHS1%) polyphenolic extract in their diet. The data show the mean ± SEM (*n* = 6). Abbreviations: NT, normal temperature; AHS, acute heat stress.

Beta diversity was accessed by unweighted and weighted UniFrac distances and analyzed through PERMANOVA. No significant variation was observed in the beta diversity in the chicken cecum among all the treatments (Figure 7).

**Figure 8:**
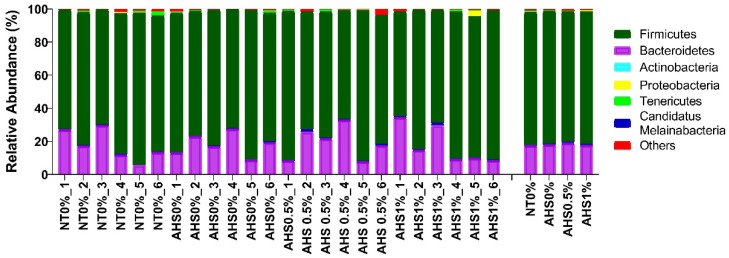
Effects of dietary polyphenol extract supplementation on the bacterial phylum composition in the chicken cecum. Chickens were fed diets containing 0% (control), 0.5%, and 1% polyphenolic extract from the 1st day to the 36th day of age. On the 37th day, birds were either kept at a thermoneutral temperature (21.0 °C) and provided with a control diet (NT0%) or heat-stressed at 31.0 °C for six hours and supplemented with 0% (AHS0%), 0.5% (AHS0.5%), and 1% (AHS1%) polyphenolic extract in their diet. The data show the mean ± SEM (*n* = 6). Abbreviations: NT, normal temperature; AHS, acute heat stress.

The two most prevalent phyla in the chicken cecum, according to a taxonomic analysis, were *Firmicutes* and *Bacteroidetes* (Figure 8). The abundance of phyla did not differ significantly (*p* > 0.05) among treatments.

**Figure 9:**
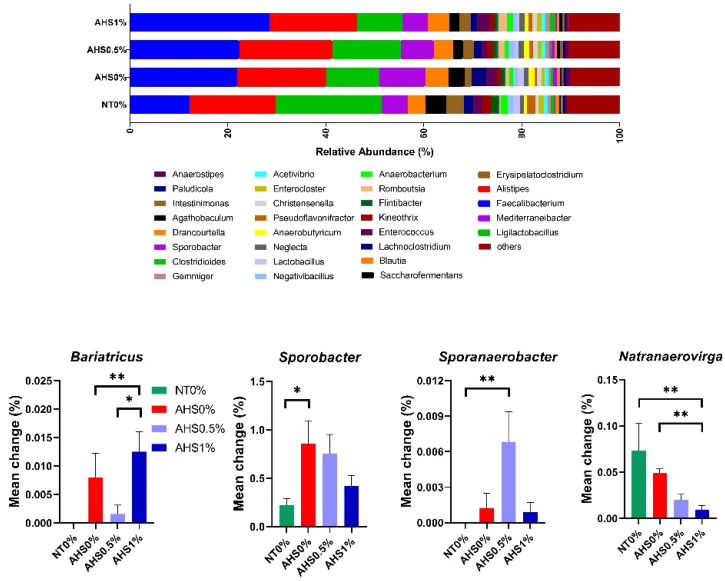
Effects of dietary polyphenol extract supplementation on the top thirty bacterial genera and the significantly modified genera such as Bariatricus, Sporobacter, Sporanaerobacter, and Natranaerovirga in the chicken cecum. Chickens were fed diets containing 0% (control), 0.5%, and 1% polyphenolic extract from the 1st day to the 36th day of age. On the 37th day, birds were either kept at a thermoneutral temperature (21.0 °C) and provided with a control diet (NT0%) or heat-stressed at 31.0 °C for six hours and supplemented with 0% (AHS0%), 0.5% (AHS0.5%), and 1% (AHS1%) polyphenolic extract in their diet. The data show the mean ± SEM (*n* = 6). * and ** indicate significant differences at *p* < 0.1 and *p* < 0.05. Abbreviations: NT, normal temperature; AHS, acute heat stress.

*Faecalibacterium*, *Alistipes*, *Ligilactobacillus*, *Mediterraneibacter*, and *Blautia* were the most abundant genera in the chicken cecum (Figure 9). The abundance of *Faecalibacterium* increased in heat-exposed birds (0% HS, 21.8%; 0.5% HS, 22.3%; 1% HS, 28.5%) compared to the thermoneutral control (12.2%). The abundance of *Ligilactobacillus* also decreased in heat-exposed birds (0% HS, 10.8%; 0.5% HS, 14%; 1% HS, 9.3%) compared to the thermoneutral control (21.7%). The abundance of *Mediterraneibacter* was increased in 0% HS (9.5%) compared to 0% NT (5.2%), 0.5% HS (6.7%), and 1% HS (5.1%).

The abundance of *Bariatricus* was higher in AHS1% than in NT0% (*p* = 0.025) and 0.5% HS (*p* = 0.1). The abundance of *Sporobacter* (*p* = 0.077) and *Sporanaerobacter* (*p* = 0.044) was higher in 0% HS and 0.5% HS, respectively, than in 0% NT. The abundance of *Natranaerovirga* was lower in 1% HS than in 0% NT (*p* = 0.022) and 0% HS (*p* = 0.009), respectively.

**Figure 10:**
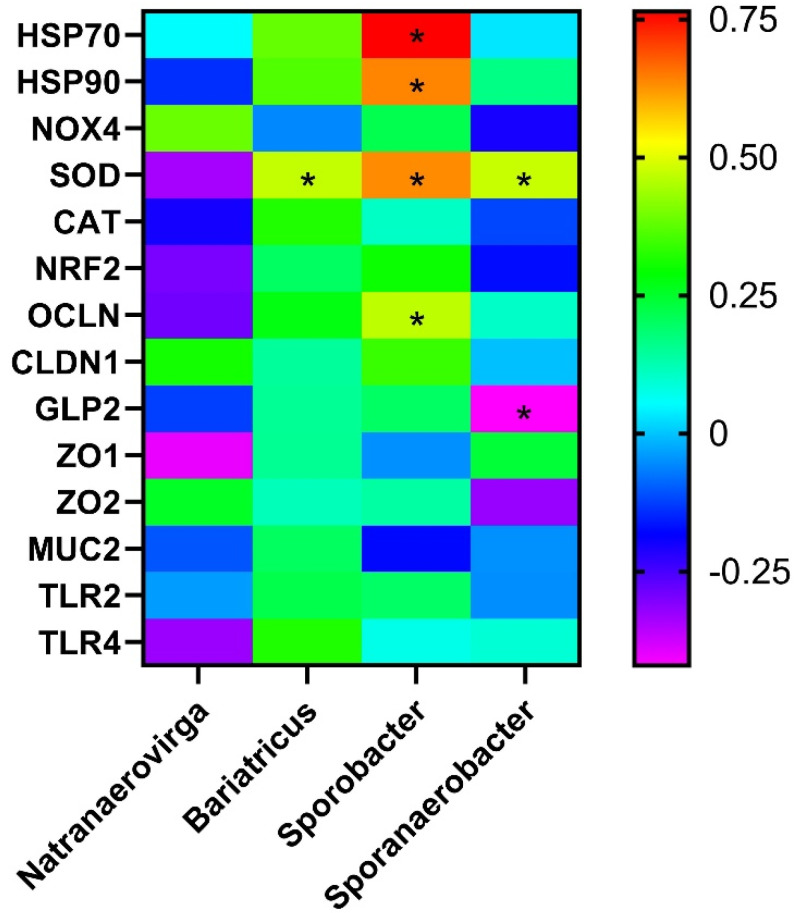
The Spearman correlation among the genes and the significantly modified bacterial genera in broilers. Zero: similarity; positive (+) values: positive correlation; negative (−) values: negative correlation; *: significant correlation (*p* < 0.05).

The Spearman correlation among the significantly modified genera and jejunal gene expression is presented in Figure 10. The abundance of the genera *Bariatricus* and *Sporanaerobacter* was positively correlated with the expression of the *SOD* gene in chickens. Similarly, the abundance of the genus *Sporobacter* was positively correlated with the expression of the *HSP70*, *HSP90*, *SOD*, and *OCLN* genes. Additionally, a negative correlation of *Sporanaerobacter* abundance with the expression of the *GLP2* gene was also seen.

## 4. Discussion

Broiler chickens are a popular protein source and, hence, one of the most important components of the livestock sector. Extensive genetic selection and nutrition programming have enabled the broilers to attain a sufficient marketable weight within 5 weeks of age. However, enhanced environmental temperatures arising due to global warming negatively influence growth performance in broilers [2,3]. This is evident in the present study where PDBW and FI were significantly decreased in the birds exposed to HS. Furthermore, a positive correlation between FBW and IBW was found, which has also been reported in our previous study [15]. Concurrently, a contrast analysis also revealed that HS had no effect on the FBW, a finding which was comparable throughout the treatments. Increased respiration rate and panting are the major driving factors for poor growth under HS conditions [4]. The birds may not be able to access feed due to enhanced respiration, reducing FI and causing a further decline in BW. In the current study, the experimental period was six hours, which might not have been enough to elicit statistical changes in FBW but in PDBW and FI.

Studies have shown that the core body temperature increases when birds are under HS [19,24,25]. Compared to birds housed at the thermoneutral temperature, heat exposure caused an increase in RT across all treatments irrespective of PSPP supplementation. However, the difference in the RT presented by ΔT showed the smallest increment in broilers supplemented with 1% PSPP compared to 0.5% PSPP (AHS0.5% vs. AHS1.0%). It might be indicative that a higher dose of PSPP may enable the bird to withstand HS for an extended time period.

Measuring organ indices could be another way to determine the effects of HS on broilers. The liver without the gallbladder is one of the giblets which is also associated with nutrition and metabolism. The bursa of the Fabricius and the spleen, on the other hand, are linked with immunity. Exposure of birds to HS may hamper the functioning of these organs and could be evaluated as modulated organ weights. However, variation in the modulation of organ weights is controlled by many external factors such as duration and intensity of heat exposure. For instance, the liver weight increased while that of lymphoid organs decreased in birds exposed to long-term HS [2,13,26,27]. Contrary to this, the absolute and relative organ weights did not differ significantly in the present study. Our results are in agreement with previous studies where acute HS had no effects on the liver and lymphoid organ weights in chickens [7,18]. The lack of variation in the absolute and relative organ weight in the current study might be attributed to the short period and low intensity of heat exposure.

Triglycerides are associated with energy production, as they are stored in the form of lipids. The concentration of triglycerides fell in HS conditions [28]. Heat-exposed birds in the current study showed significantly lower triglyceride levels (NT0% vs. AHS0%, AHS0.5%, and AHS1%; *p* = 0.067), a phenomenon which supports the aforementioned findings. After six hours of HS, the 0.5% PSPP-supplemented group had the lowest triglyceride level compared to the non-supplemented group under the thermoneutral temperature (NT0% vs. AHS0.5%; *p* = 0.037). The same group also recorded the highest (*p* = 0.001) RT and ΔT. However, the precise mechanism by which PSPP lowers triglyceride levels is unclear. It is understood that chickens tend to pant more under HS, a phenomenon which enhances respiration rates [4]. Concurrently lower triglyceride levels in the same treatment (AHS0.5%) and higher RT and ΔT indicate that the energy demand was higher due to increased panting under HS.

The HSP-related genes are reliable markers of HS, as their expression increases during heat exposure [29,30]. In the current study, the expression of the *HSP-70* and *-90* genes was increased when birds were exposed to HS. However, supplementation with 0.5% PSPP significantly reduced the expression of these HSPs compared to the AHS0% group. Hence, polyphenols might be beneficial in mitigating the effects of HS by reducing HSP expression in the jejunum. The underlying mechanism causing this is yet to be determined. However, it is expected that the reduction of the adverse effects of HS by PSPP might occur via a reduction in reactive oxygen species (ROS) generation even under HS conditions. Previous studies have elucidated the role of NOX-related genes as indicators of ROS production [31,32]. Hence, the expression of NOXs increases under HS conditions [33,34]. We evaluated the expression of the *NOX4* gene to identify the modulation of ROS production among treatments. The results of the contrast analysis revealed that HS tended to enhance the expression of the *NOX4* gene (NT0% vs. AHS0%), but the expression of the *NOX4* gene in PSPP-supplemented HS birds was similar to that in control birds kept at the thermoneutral temperature (NT0% vs. AHS0.5% and NT0% vs. AHS1%). Additionally, a lower expression of both HSP-related genes (*HSP-70* and *-90*) and the *NOX4* gene in PSPP-supplemented HS birds (AHS0.5% and AHS1%) than those in AHS0% indicates that PSPP subsides the deleterious effects of HS by reducing ROS generation.

Antioxidant enzymes play a crucial role in palliating the effects of HS. A series of enzymes activate to participate in protecting the cell by countering the ROS generation which may otherwise lead to tissue injury [11,35]. The transcription factors *NRF2* activate the production of several key genes associated with antioxidant enzymes, including *CAT* and *SOD* [36]. Under HS conditions, the expression of antioxidants has been reported to be upregulated [30]. In the current study, the expression of the *SOD* gene was higher in HS birds (AHS0%, AHS0.5%, and AHS1%) compared to the thermoneutral control (NT0%), a finding which falls in line with previously mentioned studies. The *SOD* acts as a first line of defense to counter stress [37]. Hence, an increased expression could be attributed to the increased activity to counter stress. Additionally, diets high in polyphenol-rich grape seeds increase *SOD* activity [12]. As discussed earlier, *NOX4* expression was higher in the AHS0% treatment, a phenomenon which could lead to higher ROS generation. Enhanced *SOD* gene expression in the AHS0% treatment compared to NT0% might be due to the defense against ROS generated due to HS. A further increase in the expression of the *SOD* gene in AHS0.5% and AHS1% indicates the improved antioxidant ability of the PSPP-supplemented diets.

The penetration of pathogens is controlled via intestine barrier components. However, variation in the expression of these genes in various studies has been reported. For instance, the expression of the *CLDN*, *OCLN*, *ZO1*, and *MUC2* genes was decreased when birds were exposed to HS of 10 h/d for 21 days [38]. Contrary to this, the expression of the *CLDN* and *ZO1* genes was increased when birds were heat-stressed for 8 h/d for 5 days [39]. Hence, the duration of HS could be a factor causing discrepancy in the results. In the current study, the expression of tight junctions (TJs) such as *CLDN1* and *ZO2* genes was increased in HS birds. Enhanced expression of TJ in HS birds might be due to the protective response of the birds to survive the heat exposure [40]. The possibility of the loss of function by the tight junction also exists. However, the lack of variation in most of the intestinal-related genes in PSPP-supplemented birds suggests that the detrimental effect of HS might be lowest in these treatments. Studies have shown that supplementation with phytogenic feed additives containing active phenolic components may enhance the expression of some intestinal-health-related genes in chickens [41,42,43]. Similarly, the contrast analysis results of the present study revealed a higher expression of the *ZO1* and *MUC2* genes in PSPP-supplemented birds. Thus, it can be elucidated that supplementation with PSPP may strengthen the cell junctions and mucosal layer made up of mucin, thus impairing the pathogen adhesion and penetration in the gut.

*TLRs* have idiosyncratic characteristics in imparting immunity. By recognizing pathogen-associated molecular patterns (PAMPs) and antigen-presenting cells, they are able to identify foreign particles [11]. The expression of *TLRs* is influenced by several factors depending on the severity and time of HS and the presence or absence of pathogenic microbes [44]. The expression of *TLRs* increases in a time-dependent manner [45]. The expression of *TLR2* was increased in heat-exposed birds (NT0% vs. AHS0%). Conversely, the expression of the *TLR4* gene was not affected among the same treatments, a phenomenon which might be due to the shorter heat exposure period (6 h). Supplementation with PSPP (AHS0.5%) helped overcome the enhanced expression of the *TLR2* gene. One possible reason could be the reduction in pathogenic invasion in the PSPP-supplemented group during HS. Previous studies have reported a reduction in *TLR* gene expression when pathogen-infected chickens were supplemented with polyphenols [46].

The alpha diversity was not found to be significantly affected by the AHS, a finding which is in congruence with previous reports [18]. Another study showed that the unweighted and weighted PCA were less diverse during the first day of HS but became more dispersed as the number of days under HS increased [47]. Hence, the period of HS might be a driving factor causing the paradigm microbial diversity shift. Furthermore, *Firmicutes* and *Bacteroidetes* were the two most abundant phyla in the current study. This is in congruence with previous studies [47,48]. Furthermore, *Faecalibacterium* and *Allistipes* were the two most abundant genera in the chicken cecum. *Faecalibacterium* produce butyrate that helps toughen the mucosal layer through goblet cells and mucin enrichment, thus decreasing pathogenic penetration under HS conditions [49,50]. The highest abundance of *Faecalibacterium* in the 1% PSPP-supplemented broilers might be indicative of better health and lower pathogenic invasion. Concomitantly, higher *MUC2* gene expression in the same treatment denotes the intactness of the mucosa. The abundance of *Allistipes* was almost similar across all the treatment groups.

In the present study, *Bariatricus massiliensis* was the only species detected from the genus *Bariatricus*. It is known to inhibit the histone deacetylase (HDAC) by reducing the HDAC2 isoform class 1 enzyme activity [51]. HDAC inhibition exerts an anti-inflammatory action [52] and enhances the expression of tight junction proteins [53]. This is justified in the present study, as most of the genes related to gut health and immunity showed a positive trend of correlation with the abundance of *Bariatricus* (Figure 10). An increase in the abundance of *Bariatricus* in 1% HS compared to 0% NT and 0.5% HS suggests its regenerating effects through anti-inflammation in the gut under heat exposure.

The genus *Sporobacter* may act as a pathogen in the broilers [54]. Its higher abundance is connected with pathological conditions in mice [55] and with infection in pigs [56]. In general, pathogenic conditions enhance antioxidant production to counter oxidative stress. Accordingly, the abundance of *Sporobacter* was positively correlated with the expression of antioxidant- (*SOD*) and HSP-related genes (*HSP-70* and *-90*) (Figure 10). The abundance of *Sporobacter* was significantly increased in 0% HS than in 0% NT. An increase in its abundance in the heat-exposed birds could be indicative of pathogenic penetration. Similarity in the abundance of *Sporobacter* in the thermoneutral and polyphenol-supplemented birds might be due to a lower pathogenic penetration, indicating a protective effect under HS.

*Sporanaerobacter* is a spore-forming genus that produces short-chain fatty acids (SCFAs) [57]. Previous studies have reported a positive correlation between *Sporanaerobacter* and serotonin in mice [58]. In chickens, FI is positively correlated with serotonin content [59]. In line with previous studies, the abundance of *Sporanaerobacter* was found to be negatively correlated with *GLP2* gene expression (Figure 10) in the present study. *GLP2* is known to suppress appetite [60]. Additionally, our results, in terms of the abundance of *Sporanaerobacter* and FI, showed a contradiction, as the FI was reduced in HS compared to the thermoneutral control. As explained earlier, the decrease in FI in the present study might be related to other factors such as a higher respiratory rate rather than the neurotransmitters. The lack of significant differences across treatments in the mRNA expression of *GLP2* further reinforces the stated hypothesis. Enhanced *Sporanaerobacter* abundance in 0.5% HS compared to 0% NT may help the birds to overcome the decrease in FI through neurotransmitters over time.

*Natranaerovirga* is the third most abundant genus in one-day-old chickens [61]. Contrary to this, in the current study, the abundance of *Natranaerovirga* could not be detected even in the top thirty genera. Hence, the altered findings might be due to the different age of the birds. In the present study, *Natranaerovirga pectinivora* is the only species identified and its abundance was reduced in 1% HS. Little is known about the role of the genus *Natranaerovirga* in the chicken cecum and, thus, requires further investigations.

Overall, as global warming continues to create havoc for the broiler industry, it is of the utmost importance to find effective solutions. Under challenging environmental conditions, PSPP supplementation could be a way to maintain growth performances. By improving antioxidants such as SOD, cellular damage could be lessened or prevented, leading to a better intestinal gut barrier. Further, the use of natural substances such as PSPP could be an alternative to the use of synthetic compounds for improving the health and immunity of broilers. This will not only be beneficial to the poultry farmers, but also to the consumers who prefer more sustainable poultry meat.

## 5. Conclusions

The consequences of AHS were seen in the form of reduced growth performance and enhanced RT in heat-exposed chickens. AHS enhanced the expression of stress markers, antioxidants, and gut-health-related genes and the pathogenic penetration in the caecum. Supplementation with PSPP in the diet helped suppress the stress marker genes, reduced pathogenic penetration, and enhanced the abundance of gut-favorable bacteria in broilers.

## Figures and Tables

**Table 1:** Effect of dietary polyphenol extract supplementation on the performance parameters of acute-heat-stressed (AHS) broiler chickens.

		FBW (g)	PDBW	FI (g)
Treatments	NT0%	2647 ± 41.9	0.3 ± 0.49	48.4 ± 5.39
	AHS0%	2673 ± 16.6	−2.41 ± 0.59	29.4 ± 2.84
	AHS0.5%	2598 ± 24.8	−3.02 ± 0.64	25.8 ± 1.98
	AHS1%	2619 ± 30	−1.38 ± 0.24	23.6 ± 5.04
	*p* value	0.007	0.007	0.002
IBW	Est	0.882	−0.004	0.042
	SE	0.115	0.004	0.033
	*p* value	0.001	0.347	0.227
Contrast analysis	NT0% vs. AHS0%	0.553	0.002	0.005
	NT0% vs. AHS0.5%	0.262	0.001	0.001
	NT0% vs. AHS1%	0.516	0.035	0.001
	AHS0% vs. AHS0.5%	0.096	0.413	0.541
	AHS0% vs. AHS1%	0.222	0.176	0.329
	AHS0% vs. AHS0.5%, and AHS1%	0.098	0.743	0.360
	NT0% vs. AHS0%, AHS0.5%, and AHS1%	0.624	0.001	0.001

Chickens were fed diets containing 0% (control), 0.5%, and 1% polyphenolic extract from the 1st day to the 36th day of age. On the 37th day, birds were either kept at the thermoneutral temperature (21.0 °C) and provided with a control diet (NT0%) or heat-stressed at 31.0 °C for six hours and supplemented with 0% (AHS0%), 0.5% (AHS0.5%), and 1% (AHS1%) polyphenolic extract in their diet. The data show the mean ± SEM. Abbreviations: IBW, initial body weight; FBW, final body weight; PDBW, percent difference in body weight; FI, feed intake; NT, normal temperature; AHS, acute heat stress.

**Table 2:** Effect of dietary polyphenol extract supplementation on the rectal temperature of acute-heat-stressed (AHS) broiler chickens.

	Rectal Temperature (°C)		
	Before	After	ΔT
NT0%	41.3 ± 0.08	41.3 a ± 0.07	−0.017 a ± 0.065
AHS0%	41.4 ± 0.08	43.6 b ± 0.38	2.267 bc ± 0.314
AHS0.5%	41.3 ± 0.03	44.0 b ± 0.49	2.783 c ± 0.469
AHS1%	41.2 ± 0.03	43.1 b ± 0.03	1.900 b ± 0.045
*p* value	0.317	0.001	0.001
Contrast analysis			
NT0% vs. AHS0%	0.710	0.001	0.001
NT0% vs. AHS0.5%	0.358	0.001	0.001
NT0% vs. AHS1%	0.202	0.001	0.001
AHS0% vs. AHS0.5%	0.202	0.376	0.215
AHS0% vs. AHS1%	0.106	0.256	0.374
AHS0% vs. AHS0.5%, and AHS1%	0.098	0.880	0.832
NT0% vs. AHS0%, AHS0.5%, and AHS1%	0.451	0.001	0.001

Chickens were fed diets containing 0% (control), 0.5%, and 1% polyphenolic extract from the 1st day to the 36th day of age. On the 37th day, birds were either kept at a thermoneutral temperature (21.0 °C) and provided with a control diet (NT0%) or heat-stressed at 31.0 °C for six hours and supplemented with 0% (AHS0%), 0.5% (AHS0.5%), and 1% (AHS1%) polyphenolic extract in their diet. The data show the mean ± SEM (*n* = 6). a, b, c: different letters indicate significant differences (*p* < 0.05). Abbreviations: NT, normal temperature; AHS, acute heat stress.

**Table 3:** Effect of dietary polyphenol extract supplementation on the absolute and relative organ weight of acute-heat-stressed (AHS) broiler chickens.

	Absolute (g)			Relative (%)		
	Liver	Bursa	Spleen	Liver	Bursa	Spleen
NT0%	69.94 ± 2.47	3.32 ± 0.381	2.93 ± 0.557	2.65 ± 0.158	0.13 ± 0.015	0.11 ± 0.024
AHS0%	66.03 ± 6.53	3.29 ± 0.893	3.04 ± 0.324	2.44 ± 0.18	0.13 ± 0.036	0.11 ± 0.008
AHS0.5%	59.06 ± 2.93	3.37 ± 0.438	3.75 ± 0.881	2.26 ± 0.115	0.13 ± 0.017	0.14 ± 0.035
AHS1%	67.96 ± 4.72	2.97 ± 0.272	2.77 ± 0.516	2.57 ± 0.192	0.11 ± 0.011	0.11 ± 0.02
*p* value	0.362	0.953	0.678	0.384	0.954	0.669
Contrast analysis						
NT0% vs. AHS0%	0.543	0.963	0.905	0.374	0.978	0.966
NT0% vs. AHS0.5%	0.100	0.954	0.35	0.111	0.924	0.367
NT0% vs. AHS1%	0.757	0.651	0.852	0.745	0.67	0.832
AHS0% vs. AHS0.5%	0.282	0.917	0.413	0.456	0.903	0.345
AHS0% vs. AHS1%	0.763	0.685	0.759	0.569	0.69	0.866
AHS0% vs. AHS0.5%, and AHS1%	0.649	0.861	0.765	0.918	0.873	0.651
NT0% vs. AHS0%, AHS0.5%, and AHS1%	0.291	0.857	0.721	0.249	0.883	0.789

Chickens were fed with diets containing 0% (control), 0.5%, and 1% polyphenolic extract from the 1st day to the 36th day of age. On the 37th day, birds were either kept at a thermoneutral temperature (21.0 °C) and provided with a control diet (NT0%) or heat-stressed at 31.0 °C for six hours and supplemented with 0% (AHS0%), 0.5% (AHS0.5%), and 1% (AHS1%) polyphenolic extract in their diet. The data show the mean ± SEM (*n* = 6). Abbreviations: NT, normal temperature; AHS, acute heat stress.

**Table 4:** Effect of dietary polyphenol extract supplementation on the serum biochemicals of acute-heat-stressed (AHS) broiler chickens.

	Blood Biochemicals (mg/dL)			
	Glucose	Total Protein	Cholesterol	Triglyceride
NT0%	250 ± 4.5	3.1 ± 0.2	106 ± 2.3	45.2 ± 3.9
AHS0%	283 ± 21.2	2.9 ± 0.2	112 ± 8.7	39.8 ± 8.3
AHS0.5%	272 ± 11.4	3 ± 0.1	109 ± 7.5	27.0 ± 4.1
AHS1%	275 ± 16.5	3 ± 0.1	106 ± 5.7	30.2 ± 5.6
*p* value	0.433	0.713	0.915	0.126
Contrast analysis				
NT0% vs. AHS0%	0.121	0.262	0.547	0.519
NT0% vs. AHS0.5%	0.305	0.524	0.776	0.037
NT0% vs. AHS1%	0.237	0.670	1.000	0.080
AHS0% vs. AHS0.5%	0.578	0.619	0.749	0.130
AHS0% vs. AHS1%	0.694	0.479	0.547	0.248
AHS0% vs. AHS0.5%, and AHS1%	0.584	0.488	0.594	0.125
NT0% vs. AHS0%, AHS0.5%, and AHS1%	0.128	0.373	0.717	0.067

Chickens were fed with diets containing 0% (control), 0.5%, and 1% polyphenolic extract from the 1st day to the 36th day of age. On the 37th day, birds were either kept at a thermoneutral temperature (21.0 °C) and provided with a control diet (NT0%) or heat-stressed at 31.0 °C for six hours and supplemented with 0% (AHS0%), 0.5% (AHS0.5%), and 1% (AHS1%) polyphenolic extract in their diet. The data show the mean ± SEM (*n* = 6). Abbreviations: NT, normal temperature; AHS, acute heat stress.

**Table 5:** Results of planned contrasts on the expression of various genes in the jejunum of broilers.

	NT0% vs. AHS0%	NT0% vs. AHS0.5%	NT0% vs. AHS1%	AHS0% vs. AHS0.5%	AHS0% vs. AHS1%	AHS0% vs. AHS0.5% and AHS1%	NT0% vs. AHS0%, AHS0.5% and AHS1%
HSP70	0.001	0.451	0.392	0.003	0.003	0.001	0.024
HSP90	0.001	0.303	0.057	0.013	0.085	0.018	0.011
NOX4	0.024	0.905	0.946	0.031	0.021	0.012	0.322
SOD	0.011	0.011	0.002	0.989	0.505	0.705	0.001
CAT	0.460	0.789	0.063	0.317	0.237	0.912	0.328
NRF2	0.168	0.134	0.162	0.843	0.983	0.897	0.082
OCLN	0.807	0.970	0.727	0.778	0.917	0.918	0.820
CLDN1	0.002	0.860	0.214	0.004	0.039	0.005	0.057
GLP2	0.317	0.830	0.192	0.429	0.749	0.783	0.302
ZO1	0.924	0.237	0.030	0.203	0.025	0.043	0.174
ZO2	0.122	0.113	0.540	0.004	0.037	0.005	0.789
MUC2	0.925	0.971	0.056	0.896	0.047	0.208	0.431
TLR2	0.021	0.213	0.011	0.237	0.670	0.668	0.013
TLR4	0.333	0.014	0.102	0.102	0.449	0.167	0.038

Abbreviations: HSP70, heat shock protein 70; HSP90, heat shock protein 90; NOX4, nicotinamide adenine dinucleotide phosphate oxidase 4; SOD, superoxide dismutase; CAT, catalase; NRF2, nuclear factor erythroid 2-related factor 2; OCLN, occludin; CLDN1, claudin-1; GLP2, glucagon-like peptide-2; ZO1, zonula occluden 1; ZO2, zonula occluden 2; MUC2, mucin 2; TLR2, toll-like receptor 2; TLR4, toll-like receptor 4; NT, normal temperature; AHS, acute heat stress.

## Data Availability

The original contributions presented in this study are included in the article/Supplementary Material. Further inquiries can be directed to the corresponding author.

## Supplementary Materials

The following supporting information can be downloaded at: https://www.mdpi.com/article/10.3390/microorganisms13020235/s1, Table S1: Feed composition and nutrient levels of the diet; Table S2: Primer sequences were used to evaluate the jejunal mRNA expression in broilers.
