# Supplementary material for: Alleviation of Acute Heat Stress in Broiler Chickens by Dietary Supplementation of Polyphenols from Shredded, Steam-Exploded Pine Particles
Source: Microorganisms. 2025 Jan 22;13(2):235. doi: 10.3390/microorganisms13020235 (PMC11858311; doi:10.3390/microorganisms13020235)
Supplement: Supplementary file 1 [file microorganisms-13-00235-s001.zip › microorganisms-3430193-supplementary.pdf]

Supplementary Table S1. Feed composition and nutrient levels of the diet*

Ingredients (%)	Starter<br>0-7 days	Grower<br>8-21 days	Finisher<br>22~ days
Corn	38.97	45.87	39.68
Wheat	15.00	15.00	25.00
Soybean meal (42.6% CP)	32.00	25.60	20.60
Corn gluten	3.00	2.64	3.00
Meat and bone meal	2.00	2.00	2.50
Animal fat	4.00	3.88	4.54
Salt	0.25	0.25	0.25
Tricalcium phosphate	1.30	1.04	0.86
Limestone	1.26	1.22	1.26
Sodium bicarbonate	0.00	0.02	0.00
L-Threonine	0.12	0.16	0.16
Lysine	1.23	1.44	1.32
D-L-Methionine	0.33	0.03	0.03
Choline chloride (50%)	0.03	0.03	0.03
Premix#	0.20	0.20	0.20
Phytase	0.05	0.05	0.05
Feed additive	0.25	0.25	0.25
Anti-coccidia	0.01	0.01	0.01
Calculated nutrients			
Crude protein (%)	23.00	20.50	19.50
Crude fat (%)	6.31	6.36	6.90
Crude fibre (%)	3.01	2.80	2.68
Crude ash (%)	5.99	5.34	5.02
Calcium (%)	1.01	0.90	0.86
Available phosphorous (%)	0.60	0.53	0.49

Digestible lysine (%)	1.43	1.24	1.09
Digestible methionine + cystine (%)	1.07	0.95	0.86
Copper (ppm)	82.21	81.04	80.78
Zinc (ppm)	100.27	96.63	97.33
Metabolizable energy (kcal/kg)	3050	3150	3200

* Feed was procured from Nonghyup Feed (Seoul, Korea).

[#] Trace minerals and vitamins provided in premix: Vitamin A, 12,000,000 IU; Vitamin D_{3}, 3,000,000 IU; Vitamin E, 40,000 IU; Vitamin K_{3}, 2,000 IU; Vitamin B_{1}, 2,000 mg; Vitamin B_{2}, 5,000 mg; Vitamin B_{6}, 3,000 mg; Vitamin B_{12}, 20 mg; Niacin, 40,000 mg; Pantothenic acid, 10,000 mg; Folic acid, 1,000 mg; Iron, 88,000 mg; Copper, 72,600 mg; Zinc, 60,000 mg; Manganese, 66,000 mg; Iodine, 990 mg; Selenium, 220 mg; Cobalt, 330 mg

Supplementary Table S2: Primer sequences were used to evaluate the jejunal mRNA expression in broilers.

Gene	Sequence	Accession number
ZO1	F: AAGTGGGAAGAATGCCAAAA<br>R: GGTCCTTGGATCCCGTATCT	XM_015278981.2
ZO2	F: GCCCAGCAGATGGATTACTT<br>R: TGGCCACTTTTCCACTTTTC	XM_025144669
GLP2	F: CGTGCCACAGCCATTCTTA<br>R: AGCGGCTCTGCAAATGATTA	NM_001163248.1
MUC2	F: ATTGAAGCCAGCAATGGTGT<br>R: TTGTTGGCCTTGTCATCAAA	JX284122.1
CLDN1	F: GGTGAAGAAGATGCGGATGG<br>R: TCTGGTGTTAACGGGTGTGA	NM_001013611
OCLN	F: ACGGCAAAGCCAACATCTAC<br>R: ATCCGCCACGTTCTTCAC	NM_205128.1
HSP70	F: GCTGAACAAGAGCATCAATCCA<br>R: CAGGAGCAGATCTTGCACATTT	AY143693.1
HSP90	F: CCCGAGCAAGCTGGATTCT<br>R: GGTCATCCCTATGCCGGTATC	NM_001109785
NOX4	F: CCTCTGTGCTTGTACTGTGTAG<br>R: GACATTGGAGGGATGGCTTAT	NM_001101829.1
CAT	F: ACCAAGTACTGCAAGGCGAA<br>R: TGAGGGTTCCTCTTCTGGCT	NM_001031215.1
SOD	F: AGGGGGTCATCCACTTCC<br>R: CCCATTTGTGTTGTCTCCAA<br>R: GTGGACGCTGGGATGATGTT	NM_205064.1
NRF2	F: CAGAAGCTTTCCCGTTCATAGA<br>R: GACATTGGAGGGATGGCTTAT	NM_205117
TLR2	F: GATTGTGGACAACATCATTGACTC<br>R: AGAGCTGCTTTCAAGTTTTCCC	XM_001232192294a
TLR4	F: AGTCTGAAATTGCTGAGCTCAAAT<br>R: GCGACGTTAAGCCATGGAAG	NM_001030693.1190a
GAPDH	F: TTGGCATTGTGGAGGGTCTTA<br>R: GTGGACGCTGGGATGATGTT	NM_204305.1
β-actin	F: ACCGGACTGTTACCAACA<br>R: GACTGCTGCTGACACCTT	NM_205518.1

Abbreviations: *ZO1*: Zonula occluden 1; *ZO2*: Zonula occluden 2; *GLP2*: Glucagon-like peptide-2; MUC2: Mucin 2; CLDN1: Claudin 1; OCLN: Ocluddin; HSP70: Heat shock protein 70; *HSP90*: Heat shock protein 90; *NOX4*; Nicotinamide adenine dinucleotide phosphate oxidase 4; *CAT*: Catalase; *SOD*: Superoxide dismutase; NRF2: nuclear factor erythroid 2-related factor;

TLR 2: Toll-like receptors 2; TLR 4: Toll-like receptors 4; *GAPDH*: Glyceraldehyde-3-phosphate dehydrogenase.
